# Prediction Equations for Body-fat Percentage in Indian Infants and Young Children Using Skinfold Thickness and Mid-arm Circumference

**DOI:** 10.3329/jhpn.v28i3.5548

**Published:** 2010-06

**Authors:** Bandana Sen, Kaushik Bose, Saijuddin Shaikh, Dilip Mahalanabis

**Affiliations:** ^1^ Society for Applied Studies, CF-198, Salt Lake City, Sector I, Kolkata 700 064, India; ^2^ Department of Anthropology, Vidyasagar University, Paschim Midnapore 721 102, India

**Keywords:** Anthropometry, Body mass index, D_2_O dilution, Fat-mass, Infants, Mid-arm circumference, India

## Abstract

The objective of the study was to develop prediction equations for fat-mass percentage in infants in India based on skinfold thickness, mid-arm circumference, and age. Skinfold thicknesses and mid-arm circumference of 46 apparently-healthy infants (27 girls and 19 boys), aged 6–24 months, from among the urban poor attending a well baby clinic of a hospital in Kolkata were measured. Their body-fat percentage was measured using the D_2_O dilution technique as the reference method. Equations for body-fat percentage were developed using a stepwise forward regression model using skinfold thicknesses, mid-arm circumference, and age as independent variables, and the body-fat percentage was derived by D_2_O dilution as the dependent variable. The new prediction equations are: body-fat percentage=-69.26+5.76×B-0.33×T^2^+5.40×M+0.01×A^2^ for girls and body-fat percentage=-8.75+3.73×B+2.57×S for boys, where B=biceps skinfold thickness, T=triceps skinfold thickness, and S=suprailiac skinfold thickness all in mm, M=mid-arm circumference in cm, and A=age in month. Using the D_2_O dilution technique, the means (SD) of the calculated body-fat percentage were 17.11 (7.25) for girls and 16.93 (6.62) for boys and, using the new prediction equations, these were 17.11 (6.25) for girls and 16.93 (6.02) for boys. The mean of the differences of paired values in body-fat percentage was zero. The mean (SD) of the differences of paired values for body-fat percentage derived by the D_2_O technique and the new equations, applied on an independent sample of 23 infants (11 girls and 12 boys) were -0.93 (6.56) for girls and 1.14 (2.43) for boys; the 95% confidence limits of the differences of paired values for body-fat percentage were -2.03 to +3.89 for girls and -0.26 to +2.54 for boys. Given that the trajectories of growth during infancy and childhood are a major risk factor for a group of diseases in adulthood, including coronary heart disease and diabetes, these predictive equations should be useful in field studies.

## INTRODUCTION

Studies in developed countries have shown that slow growth during the first two years of life is associated with an increased risk of coronary heart disease in adults, independently of birthweight (1–4). However, postnatal rapid gain in weight has also been suggested to be a risk factor for later obesity, elevated blood pressure in adolescent males, impaired glucose tolerance in young adults, and increased mortality from coronary heart disease (5–11). A recent analysis of longitudinal data in low-income and middle-income countries has shown that stunting in the first two years of life leads to shorter height in adults, lower attained schooling, and decreased birthweight of offspring (12). The analysis has also shown that stunting between 12 and 36 months of age predicts poor cognitive performance and/or lower school grades attained in middle childhood (12). Further, published work from high-income countries suggests a consistent association between intrauterine growth restriction and an increased risk of several chronic diseases in adulthood, such as coronary heart disease, diabetes, and hypertension (13–16).

In light of these findings, precise measurement of body composition, such as fat-free mass (FFM) and fat-mass (FM) in infancy and early childhood, assumes importance. Many methods for measuring body-fat percentage are sophisticated and only suitable for the research setting (17–22). Among the large number of body-composition assessment methods in common use, anthropometry, including skinfold measurements, is simple, portable, and cost-efficient field methods.

We need a technique that provides reliable and valid estimates of body-fat in infants and young children that are non-invasive and are suitable for use in the community and outside fixed facilities. Sufficiently accurate measurement of length of infants and young children for use in equations for body composition is difficult to carry out in the field, largely because portable measuring instruments are inaccurate and one needs the child's cooperation. Similarly, for measuring weight, one needs to carry weighing scales to the field. On the other hand, with adequate training, health workers can measure mid-arm circumference and skinfold thickness with portable equipment and with less burden on participants. In the present study, we developed two new equations for estimating the body-fat percentage (one for girls and the other for boys) on 6–24-month old infants using skinfold thickness (biceps, triceps, and suprailiac), mid-arm circumference, and age. We used the D_2_O dilution technique as the reference method. We also validated these equations on an independent sample of infants to estimate the body-fat percentage and compared them with the estimates derived by the reference method.

## MATERIALS AND METHODS

### Subjects

The study was conducted among the urban poor children attending a well baby clinic of a large charitable government hospital in the city of Kolkata, India. Sixty-nine children, aged 6–24-months, participated in the study. The socioeconomic and demographic features of the families are presented in Table 1. The equations were developed on 46 (27 girls and 19 boys) consecutively-enrolled infants and young children, and then these predictive equations were validated on 23 infants (11 girls and 12 boys) in the same age-group and from the same community enrolled for this purpose. It may be noted that regression equations fitted on a sample can only be validated on an independent sample. The sample-size was largely determined by the funds made available by the Society for Applied Studies Trust for this research project. Written informed consent was obtained from their parents, and anonymity of the subjects was strictly preserved. The prediction groups of infants were initially recruited, and the prediction equations were developed. The validation group was recruited after that. The plan was to recruit a larger number for validation. High expense for the D_2_O test restricted the numbers.

**Table 1.** Characteristics of study subjects on whom prediction equations were developed and validated

Variable	Prediction group*		Validation group§	
	Girls (n=27) Mean±SD	Boys (n=19) Mean±SD	Girls (n=11) Mean±SD	Boys (n=12) Mean±SD
Age (months)	15.9±6.08	13.6±5.62	16.9±4.69	19.0±4.51
Weight (kg)	8.8±1.63	8.6±1.10	8.6±1.17	9.8±1.25
Length (cm)	75.8±6.98	75.0±5.69	75.8±4.92	80.3±4.72
BMI (percentile)	28.9±18.8	26.6±28.2	27.5±24.2	32.0±31.02
MAC (cm)	13.9±0.82	14.3±1.05	13.9±0.95	14.1±0.20
Biceps (mm)	3.4±0.78	3.5±0.95	3.3±0.68	3.17±0.72
Triceps (mm)	5.8±0.99	5.4±1.40	5.0±0.87	5.5±1.00
Suprailiac (mm)	4.6±1.02	4.9±1.39	4.2±0.75	4.3±1.39
WHZ	-1.0±0.62	-1.3±1.02	-1.3±0.79	-1.3±1.20
WAZ	-1.3±1.01	-1.4±0.98	-1.7±0.77	-1.5±1.21
HAZ	-0.77±1.37	-0.8±0.72	-1.2±0.78	-0.9±1.38

*The group of infants on whom the prediction equations were developed;

§The group of infants on whom the new equations were validated;

WHZ, WAZ, and HAZ are weight-for-length, weight-for-age and length-for-age standard deviation scores respectively compared to National Center for Health Statistics reference;

BMI=Body mass index;

MAC=Mid-arm circumference;

SD=Standard deviation

### Measurements

We used the D_2_O dilution technique for measuring total body-water (TBW). We measured length, weight, skinfold thicknesses (biceps, triceps, and suprailiac), and mid-arm circumference of these infants (23–27).

**Length:** Recumbent length was measured with a wooden measuring-board as the age was less than two years. Two examiners were required to correctly position the infant and ensure the accurate and reliable measurement of length. The subject was placed with the face upward and the head towards the fixed end and the body parallel to the long axis of the board. The shoulder-blades were rested against the surface of the board. One examiner applied gentle traction, brought the crown of the child's head into contact with the fixed headboard, and positioned the head so that the Frankfort plane was vertical. The second examiner held the subject's feet, without shoes, toes pointing directly upward and keeping the subject's knees straight, and brought the movable footboard to rest firmly against the heels. The reading was taken to the nearest 0.1 cm. Only the left leg was positioned for the measurement when the subject was restless (9).

### Skin-fold thickness

Measurements of skinfold thickness were performed in duplicate on the left side of the body on all subjects with a Lange Skinfold Calliper (Cambridge Scientific Instruments, Cambridge, MD). All measurements were undertaken according to the Anthropometric Standardization Reference Manual (26). The methods have also been described and illustrated by Cameron (25).

**Triceps:** Vertical fold was measured at the posterior midline of the upper arm, halfway between the tip of the shoulder and tip of the elbow, the elbow remaining in an extended and relaxed position.

**Biceps:** It was measured as the thickness of a vertical fold on the front of the upper left arm and directly above the centre of the cubital fossa, at the same level as the triceps skinfold.

**Suprailiac:** It was measured in the mid-axillary line immediately superior to the iliac crest. The skinfold is picked up obliquely just posterior to the mid-axillary line and parallel to the cleavage lines of the skin (26). The suprailiac is not predominantly ‘vertical’ but rather follows the natural fold of the skin.

### Mid-arm circumference

A plastic non-elastic tape was used for measuring mid-arm circumference. The measurement was taken at the midpoint of the upper left arm, between the acromion process and the tip of the olecranon. After locating the midpoint, the left arm was extended so that it was placed loosely by the side, with the palm facing inwards. The tape was wrapped gently but firmly around the arm at the midpoint. Measurements were taken to the nearest millimetre (24).

**Weight:** Weight was measured nude, using an electronic platform balance (Avery India Limited, model no. L111A) with a precision of 10 g. The balance was checked regularly for accuracy using standard weights.

**Age:** Age was calculated as the difference between the date of study and the date of birth of the child (as shown on their birth certificates).

**D_2_O dilution method:** Deuterium oxide (D_2_O), water composed of a stable isotope of hydrogen, was used. The test was performed between 9 am and 2 pm in the study centre. Baseline saliva of children was collected using a disposable syringe without needle. The subject then drank D_2_O (99.9%, Sigma) in a dose of approximately 50 mg/kg of body-weight (23, 28). We weighed the D_2_O dose in a disposable container using a precision balance, and the weight with an accuracy of 0.1 mg was recorded and used in calculating TBW. It was then mixed with 20 mL of distilled water, and the child drank it from a disposable syringe without needle. The empty container was rinsed with two consecutive lots of 15 mL of distilled water, and the child drank both the lots from the same disposable syringe. Time was allowed to equilibrate the D_2_O into the body-fluid. No food and water was permitted during the equilibration period of three hours. At the end of the equilibration period, the follow-up saliva sample was collected and immediately stored at -20°C until transported to the St John's Hospital, Bangalore, India, for further analysis. The samples were analyzed for deuterium using a duel-inlet mass spectrometer (Europa Scientific, Crewe, UK) using the zinc reduction technique (27). The results of D_2_O concentration in the saliva samples were used for calculating the TBW using the following equation (29):

TBW in moles (N_2_)=(F_1_×N_1_)÷(F_2_×1.041), where

F_1_=dose in atom percent×10^6^/100–150 ppm

F_2_=ppm after dose-ppm before dose

N_1_=Dose in g/molecular weight of D_2_O (20.0274)

TBW in kg=(TBW in moles×molecular weight of H_2_O (18.0153))/1000

The FFM was obtained as TBW in kg divided by an age and sex-specific hydration factor for FFM, and for this age-group, it ranged from 80.7 to 77.0 for boys and 80.7 to 78.0 for girls (30).

Body-fat percentage={(Weight-FFM)/weight}×100

### Regression equations

We used the fat percentage derived from the equation based on the D_2_O dilution method as stated above for developing regression equations using a forward stepwise regression model. We fitted linear regression models with the three skinfold measurements (biceps, triceps^2^, suprailiac), mid-arm circumference, and squared values of age as predictor variables. The best fitted models with biceps and suprailiac as predictors, i.e. Model 2 for boys and Model 5 (with mid-arm circumference, age^2^, triceps^2^, biceps as the predictor variables), for girls were chosen. For comparing the models, we used adjusted R^2^ values (31). We examined the degree of agreement between the body-fat percentage derived by the new equations with those derived from the D_2_O dilution equation on the infants and young children on whom the equations were developed. For developing the equations, we have added one variable at a time as predictor variables as stated earlier and examined the values of adjusted R^2^.

### Statistical analysis

The Epi Info™ software (version 6) (32) and the Stata software (version 7.0) (33) were used. For anthropometric data, a software package based on the National Center for Health Statistics (NCHS) (Centers for Disease Control and Prevention–CDC) database as provided with the Epi Info software was used. These anthropometric calculations were based on the growth reference curves developed by the NCHS and CDC (34). The best fit equations derived by regression models were first examined on the infants on whom the equations were developed. We then used these on a new set of infants in the same age-group, who also had the D_2_O dilution test and compared the body-fat percentage derived by the developed equations and by the D_2_O reference method. The percentage of fat derived by the new equations was plotted against those based on the equation for D_2_O dilution by scattergrams, and the agreement was visually examined to note if the points lie close to the ‘line of equality’, i.e. the line at a 45° angle. Furthermore, the differences in the body-fat percentage for each child of the validation set between those derived by the new equations and by D_2_O dilution were evaluated for their deviation from zero by *t*-test. For reasonably symmetric distribution, we expect the range, mean±2 standard deviation, to include about 95% of the observations, and we used this to indicate 95% limits of agreement (31). To evaluate how well the methods are likely to agree for comparing groups, we used 95% confidence interval of the mean of the differences in each subject to examine the agreement between the two groups. For a good agreement, we expect that the range, mean of the difference ±2 standard error to indicate 95% limits of agreement within which group means will lie 95% of the time.

### Ethical issues

Ethical clearance was obtained from the Ethical Review Committee of the Society for Applied Studies, Kolkata. The objective of the study was explained, and the written informed consent was obtained from their parents.

## RESULTS

The study children came from a relatively-poor community in a metropolitan city in India. The average family income was US$ 44 per month, and 65% of the families lived in one room. Fifty-six percent of the mothers had school education of six years or more. These features are comparable with the norms for the urban population in India. The mean (SD) of age, weight, length, body mass index (BMI) percentiles for age and sex, biceps, triceps, and suprailiac skinfold thickness, mid-arm circumference and z-scores for weight-for-age, length-for-age, and weight-for-length of the infants on whom the equations were developed (i.e. prediction group: 27 girls and 19 boys) and those in whom these equations were validated (i.e. validation group: 11 girls and 12 boys) are shown in Table 1. Seventeen children (9 girls and 8 boys) in the prediction group and two children in the validation group were aged less than 12 months. Others were aged 12–24 months. Table 2 shows the adjusted R^2^ values for each model with the increasing number of predictor variables. Many other models were also examined but not shown here. We note that the last model (i.e. Model 5) was the best fit for girls. For boys, Model 2 was the best fit model. Model 5 included biceps, mid-arm circumference, age^2^, triceps^2^, Model 2 included biceps and suprailiac as predictor variables. The regression coefficients (SE) of the chosen models based on fat-mass percentage derived by D_2_O dilution as the reference method are shown in Table 3. We have taken these values to construct the final equations, which are:

**Table 2.** Evaluated regression models based on fat percentage derived by D_2_O dilution method as dependent variable

Model	Girls	Boys
Independent variable	Adjusted R^2^	Adjusted R^2^
Biceps*	0.17	0.55
Biceps, suprailiac*	0.14	0.80
Biceps, (triceps^2^)*	0.14	0.61
Biceps, triceps^2^, MAC†	0.64	0.60
Biceps, triceps^2^, MAC, (age^2^)§	0.70	0.62

*Skinfold thickness in mm;

†Mid-arm circumference in cm;

§Age in months;

MAC=Mid-arm circumference

**Table 3.** Regression coefficients with standard errors of independent variables for chosen models

Boys*		Girls†	
Variable	RC (SE)	Variable	RC (SE)
Biceps**	3.73 (0.81)	Biceps**	5.76 (1.55)
Suprailiac**	2.57 (0.55)	MAC††	5.40 (1.22)
Y-intercept	-8.75 (3.03)	(Triceps2)**	-0.33 (0.11)
		(Age2)§	0.012 (0.005)
		Y-intercept	-69.25

*Adjusted R^2^=0.80;

†Adjusted R^2^=0.70;

**Skinfold thickness in mm;

††Mid-arm circumference in cm;

§Age in months;

RC=Regression coefficients;

SE=Standard error

Girls: body-fat percentage=-69.26+5.76×B -0.33×T^2^+ 5.40×M+0.01×A^2^ (1)

Boys: body-fat percentage=-8.75+3.73×B+2.57×S (2), where

B=biceps skinfold thickness in mm, T=triceps skinfold thickness in mm,

S=suprailiac skinfold thickness in mm, M=mid-arm circumference in cm, and A=age in months.

The mean (SD) of the body-fat percentage calculated by the new equations was 17.11 (6.25) and 16.93 (6.02) for girls and boys respectively. Likewise, the mean (SD) of the body-fat percentage calculated by the D_2_O dilution methods was 17.11 (7.25) and 16.93 (6.62) for girls and boys respectively. The 95% confidence limits of the mean of the difference in the body-fat percentage derived by D_2_O and by the new equations were -1.45 to +1.45% for girls and -1.33 to +1.33% for boys. As expected, the mean of the difference in the body-fat percentage was zero.

### Applicability of predictive equations

**Fig. 1.**
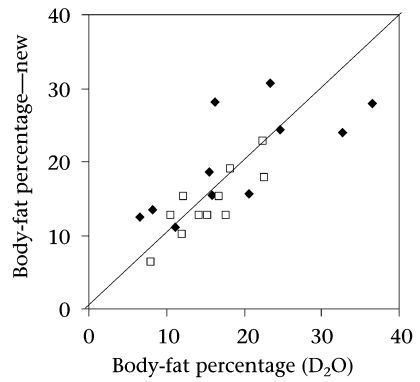
Individual data-points of body-fat percentage comparing values derived by group of girls (♦) and boys (□) under angle) the reference method (D_2_O dilution) with new predictive equations for by the reference method (D_2_O dilution) with new predictive equations for validation study along the ‘line of identity’ (at 45° angle)

**Fig. 2.**
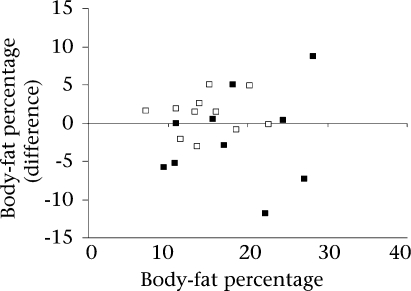
Bland-Altman plots of the difference between body-fat percentage derived by D_2_O reference method and by new equations for validation group of girls (▪) and for boys (□) plotted against average body-fat percentage derived by D_2_O method and respective new equations a: mean difference=-0.93, SD=6.56 b: mean difference=1.14, SD=2.43

Using the new prediction equations (1 and 2), we calculated the fat percentage in an independent sample of 23 infants (11 girls and 12 boys) in the same age-group and from the same community. We also calculated the body-fat percentage on them using the D_2_O dilution equation. The mean (SD) of the predicted body-fat percentage derived by the new equations was 20.15 (7.06) and 14.21 (4.27) and by the D_2_O dilution was 19.22 (9.54) and 15.36 (4.49) for girls and boys respectively. The mean (SE) of the difference in the body-fat percentage was -0.93 (1.98) for girls and 1.14 (0.70) for boys. The mean (SD) for the difference in the body-fat percentage was -0.93% (6.56) for girls and 1.14% (2.43) for boys. Distribution of data points along the line of identity (45°) between the body-fat percentage derived from the D_2_O dilution method and by the new equation applied on the validated group is shown in Figure 1. The Bland-Altman plots of the paired differences between the body-fat percentage derived by the D_2_O reference method and the new equations for the validation group of girls and boys are plotted against average body-fat percentage derived by the D_2_O method and respective new equations (Fig. 2) (35).

SD=Standard deviation

**Fig. 3.**
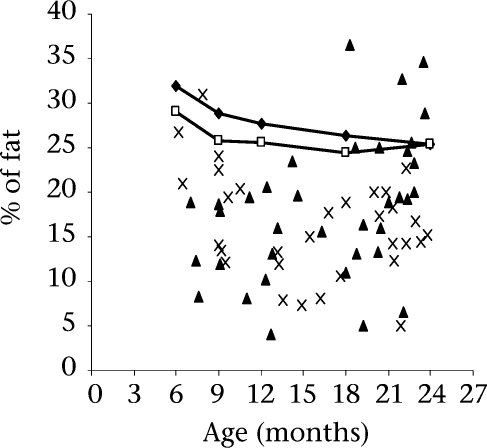
Individual data-points for body-fat percentage derived by D_2_O dilution reference method (boys ×, girls ▴) are plotted against age. The connected lines represent the mean body-fat percentage for age of healthy American infants (Boys 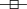, Girls 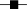) of the same age-range derived by multicomponent models (30)

## DISCUSSION

The purpose of this study was to develop predictive new equations for body-fat percentage using simple measurements, such as skinfold thickness and mid-arm circumference, for use in community nutritional studies of infants and young children in India. For infants and young children, a few regression equations to estimate the body-fat percentage derived from density are available (36). The relationship between total body-density and skinfold thickness varies with age and sex (37–39). Weststrate and Deurenberg, however, used age and sex-specific equations based on skinfold thickness to derive body-density and body-fat percentage (40). In the infants we studied, the calculated body-fat percentage based on these equations (data not shown) was substantially lower than the ones derived by the D_2_O dilution reference method and, as such, not suitable for this population.

We were able to validate these new equations on a small number of children and to show that the mean difference of the body-fat percentage was only-0.93 for girls and 1.14 for boys. In South Asian countries, there are no equations of this type for this age-group (6–24 months) for calculating the body-fat percentage. Our results showed that the proposed equations might be used for deriving the percentage of body-fat of infants to compare among groups. The number of children for validation was relatively small, which was largely limited by the cost of D_2_O estimation. In the absence of any validated equations for infants in South Asia, these equations should, therefore, be useful until further validation is feasible with additional data. Due to relatively wide limits of agreement (i.e. relatively large standard deviation of the mean difference) with the reference method, these equations are less suited for the assessment of body-fat of individual infants. However, the 95% confidence limits of the mean difference of paired values in the validation set of the infants suggest that these equations should be useful for public-health and epidemiological studies.

The measurement of skinfold thicknesses at conventional sites may not capture some important components of body-fat, i.e. central fat and lower limb-fat, in infants. We speculate that adding measurements, such as waist-circumference, thigh- and calf-skinfolds and circumferences, may improve the limits of agreement for body-fat percentage with those derived by the reference methods. Substantial changes in body-composition occur during infancy and puberty. Thus, assessment of body-composition in infants is more challenging than in adults. Barring cadeveric studies, measurements of body-composition are indirect and involve assumptions that may introduce error. Prediction equations based on anthropometric measures are derived from samples of healthy children and are dependent on several important assumptions, such as water and mineral composition of FFM, fat distribution, and patterns of skeletal maturation. Skinfold thicknesses, particularly triceps, are also directly used as indicators of fatness. Reference data exist for individuals from one year of age through adulthood (40, 41). The sum of triceps and subscapular skinfolds is also considered to be a good indicator of overall fatness, and reference data are available (41–43). To use skinfold thickness directly to indicate fatness or leanness, we need reference data for infants which are not available.

To compare the body-fat percentage in the study infants with those of infants of similar age in developed countries, we plotted individual data-points of body-fat percentage derived by D_2_O dilution in all the study infants against the mean reference data (Fig. 3) on healthy American infants of similar age derived by multi-component modelling (30). We note that the body-fat percentage in both boys and girls are generally much lower than those found in healthy American infants of similar age-group. These apparently-healthy infants drawn from a relatively low socioeconomic stratum in a metropolitan city in India are generally very lean. This phenomenon largely reflects inadequate nutrition during infancy and early childhood and is likely to be a consequence of the well-known phenomenon of prolonged breastfeeding combined with inadequate weaning food of low energy-density.

**Fig. 4.**
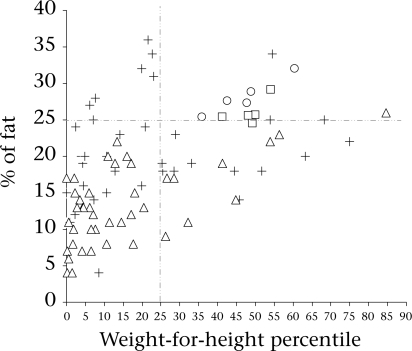
Individual data-points for body-fat percentage derived by D_2_O dilution reference method (boys ▵, girls +) are plotted against the weight-for-height percentile. The mean body-fat percentage of American infants (boys □, girls ˚) of the same age-group derived by multicomponent models are plotted against their weight-for-height percentiles (30)

Babies in South Asia have a lower birthweight than their Western counterparts, and studies have shown that body-fat is relatively preserved in these babies leading to their description as ‘thin-fat’ (44). Further, adult populations in India have a higher percentage of body-fat for a given BMI than Western populations (45, 46). Based on the above, it has been speculated that increased body-fat at birth tracks into adulthood depending on environmental constraints (47). This study has generated quality data on fat and lean body-mass percentage in a group of infants from low- to middle-income families using a reference method based on the D_2_O dilution technique. On examining the association of the percentage of fat in our study infants (Fig. 4) with leanness as indicated by their weight-for-length percentile, we note that 52 (83%) are below 25^th^ percentile for weight-for-length by the NCHS standard who may be considered lean. Of these 52 infants, six (11.5%) had more than 25% body-fat. Therefore, nearly 12% of the lean infants had high fat percentage who may be considered to fall among the so-called thin-fat phenotype as described by Yajnik and colleagues (44). In a critical evaluation of the phenomenon of thin-fat phenotype at birth, Muthayya and colleagues have shown that the thin-fat paradigm may not apply for babies small-for-gestational age (47). Since the birthweights of the study infants are not known, approximately 30% of the babies born in this population are known to have low birthweight (48)

The equations we have developed for calculating fat percentage in infants and young children will be useful in studying growth and body-composition in the first two years of life. Studying body-composition in this age-group has some relevance. There is good evidence that the trajectories of growth during infancy and childhood are a major risk factor for a group of chronic diseases that include coronary heart disease and diabetes (1–4, 49). Although low birthweight is known to be associated with increased rates of coronary heart disease and related disorders, such as stroke, hypertension, and type 2 diabetes in adulthood, recent findings indicate that impaired growth in infancy and rapid gain in the childhood weight are independently associated with these chronic diseases of adults (1). More specifically, low BMI at two years of age and high BMI at 11 years of age were shown to be associated with coronary events. On the other hand, there is evidence to suggest that faster growth as indicated by percentile crossing, particularly in infancy is associated with metabolic syndrome in later life (50).

## ACKNOWLEDGEMENTS

The work was supported by a grant from the Society for Applied Studies Trust.

The authors thank Dr. Anura V. Kurpad for doing the D_2_O estimation in the saliva samples at his laboratory at St. Johns Medical College, Bangalore. They also thank Md. Jakir Hossain for statistical assistance.
